# 20-Hydroxy-3-Oxolupan-28-Oic Acid, a Minor Component From *Mahonia bealei* (Fort.) Carr. Leaves Alleviates Lipopolysaccharide-Induced Inflammatory in Murine Macrophages

**DOI:** 10.3389/fbioe.2021.701876

**Published:** 2021-06-17

**Authors:** Xiaojun Yang, Jing Zhou, Yang He, Lingyun Lv, Yufeng Cao, Weicheng Hu

**Affiliations:** ^1^College of Food Science and Pharmacology, Xinjiang Agricultural University, Ürümqi, China; ^2^Jiangsu Collaborative Innovation Center of Regional Modern Agriculture and Environmental Protection, School of Life Sciences, Huaiyin Normal University, Huai’an, China; ^3^Department of Otorhinolaryngology-Head and Neck Surgery, The Affiliated Huaian No.1 People’s Hospital of Nanjing Medical University, Huai’an, China

**Keywords:** 20-hydroxy-3-oxolupan-28-oic acid, anti-inflammatory, NF-κB, transcriptome analyses, *Mahonia bealei*

## Abstract

20-Hydroxyl-3-oxolupan-28-oic acid (HOA), a minor component from *Mahonia bealei* (Fort.) Carr. leaves, has been found to attenuate inflammatory responses. However, the underlying molecular mechanism is still unclear. In this study, we performed a comprehensive transcriptional study to investigate genetic changes. We used RNA sequencing technology to analyses the transcriptional changes in RAW 264.7 cells in a control group, lipopolysaccharide (LPS)-induced group, and HOA-treated group. We identified 1,313 and 388 differentially expressed genes (DEGs) in the control/LPS group and LPS/HOA group, respectively. Gene Ontology (GO) classification revealed that the DEGs were mainly enriched in a series of inflammatory and immune-related processes. The results of Kyoto Encyclopedia of Genes and Genomes (KEGG) pathway analyses showed that the DEGs were mainly enriched in inflammatory-related pathways such as the nuclear factor-kappa B (NF-κB) signaling pathway, cytokine-cytokine receptor interaction, chemokine signaling pathway, mitogen-activated protein kinase (MAPK) pathway, and Janus kinase-signal transducer and activator of transcription proteins (JAK-STAT) signaling pathway. The results of qPCR validation revealed that dynamic changes in immune-related mRNAs such as Saa3, Bcl2l1, Mapkapk2, Ccl9, Sdc4, Ddx3x, Socs3, Prdx5, Tlr4, Lif, IL15, Tnfaip3, Tet2, Tgf-β1, and Ccl20, which were significantly upregulated in the LPS group and downregulated in the HOA group. Taken together, these results suggest that HOA may be used as a source of anti-inflammatory agents as well as a dietary complement for health promotion.

## Introduction

Inflammation, as a defense system, is an extremely important process that protects the body from bacterial, viral, and fungal infections (Guinane and Cotter, 2013). The moderate inflammatory response can remove foreign body antigens, necrotic tissue, and injury factors, and an appropriate number of inflammatory factors can participate in the regeneration of damaged tissues, which are conducive to wound repair and healing (Pappas et al., 2013). However, excessive inflammatory reactions can destroy normal tissues and cells, and induce various inflammatory diseases such as atherosclerosis, cardiovascular disease, sepsis, diabetes, and arthritis (Pan et al., 2010; Du et al., 2019). The spread of inflammation can further induce an acute systemic inflammatory response. Macrophages play a crucial role in the occurrence, maintenance, and elimination of inflammation. When stimulated, macrophages produce a variety of cytokines and inflammatory-related enzymes, such as interleukin-1 (IL-1β), interleukin-6 (IL-6), tumor necrosis factor-α (TNF-α), and nitric oxide (NO) (Hu et al., 2016a; Li et al., 2016, 2019). Inhibiting the excessive production of these inflammatory mediators may play a beneficial role in inflammatory diseases.

With the development of second-generation sequencing technology, transcriptomics has entered a period of rapid development. RNA sequencing (RNA-seq) technology can comprehensively and deeply record the sequences of mRNA, small RNA, and non-coding RNA (ncRNA). Compared to the traditional hybrid sequencing method, RNA-seq can reveal the exact location of the transcriptional boundary and achieve single-base resolution (Levy and Myers, 2016). It can also describe the multi-functional elements such as exons, introns, and transcriptional initiation sites in the genome, and display the sequence changes in the transcriptional region (Kukurba and Montgomery, 2015). In addition, new transcripts and splice sites can be found, and gene expression and differential expression analyses of transcripts under different conditions can be quantified. Finally, the functions of non-coding regions, such as microRNA, non-coding long RNA, and RNA editing, can be discussed (Wang et al., 2016). The application of this technology can provide more rapid and accurate biological transcription data in humans. RNA-seq has been used to study the transcriptomes of *Arabidopsis thaliana*, mice, and humans (Zhang et al., 2019).

*Mahonia*, a flowering evergreen tree of the family Berberidaceae has been reported to show a variety of biological activities, including anti-bacterial, antioxidant, anti-proliferation, and anti-inflammatory effects (Hu et al., 2011). In previous work, we isolated a lupane-type triterpene, 20-hydroxy-3-oxolupan-28-oic acid (HOA), from the dichloromethane fraction of *Mahonia bealei* (Fort.) Carr leaves, which has an obvious anti-inflammatory effect in RAW 264.7 cells (Hu et al., 2016b; Cao et al., 2019). However, the precise mechanism of its action is still unclear. In this study, RNA-seq was used to detect genomic changes induced by HOA in lipopolysaccharide (LPS)-stimulated RAW 264.7 cells. The sequencing results lay a foundation for further studies of the anti-inflammatory mechanism of HOA.

## Materials and Methods

### Materials

Experiments were performed with the RAW 264.7 cell line, obtained from the American Type Culture Collection (Rockville, MD, United States). TRIzol reagent used to separate RNA was purchased from Ambion (Austin, TX, United States). The fetal bovine serum (FBS) was from Corning (Medford, MA, United States). The RPMI1640 medium was from Gibco BRL (Life Technologies, China). Penicillin-streptomycin solution (10,000 unit/10,000 μg/mL) was purchased from Invitrogen-Gibco (Carlsbad, CA, United States). SYBR real-time PCR kit was obtained from Bio-Rad (Hercules, CA, United States).

### Extraction and Isolation

**FIGURE 1:**
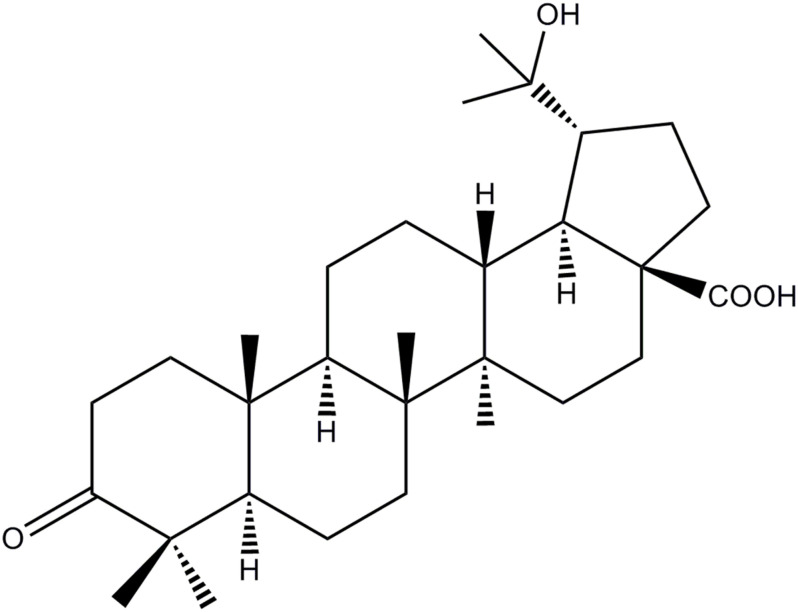
Chemical structure of HOA.

The HOA used in the experiment was separated from the leaves of *M. bealei* (Figure 1). Briefly, the active fraction was prepared as our pervious literature under the active-guide isolation way (Hu et al., 2016b). Forty-gram of CH_2_Cl_2_ fraction was then subjected to a silica gel with a gradient solvent system of hexane/CH_2_Cl_2_ (5:1-1:2) affording 11 fractions based on TLC analysis. Fra 8 was further purified by preparative HPLC to afford HOA (21.0 mg) and the purity of HOA was about 97% determined by high performance liquid chromatography (HPLC).

### RAW 264.7 Cells Culture and HOA Treatment for RNA-Seq Experiment

RAW 264.7 cells were maintained in RPMI 1640 medium supplemented with 10% FBS and 1% antibiotics (v/v) at 37°C in 5% CO_2_ atmosphere. For the RNA-Seq and RT-qPCR validation experiments, cells were pre-treated with 40 μg/mL HOA for 30 min and then incubated with LPS (1 μg/mL) for 6 h. Total RNA was extracted using TRIzol reagent according to the manufacturer’s protocol.

The quantitative and qualitative analysis of RNA was carried out with 1% agarose gel, and the degradation and pollution of RNA were monitored. The purity of RNA was determined by the Nano-Drop One (Thermo Fisher Scientific, Waltham, MA, United States). RNA concentration was measured using Nano and the integrity was assessed using the RNA Nano 6000 Assay Kit of the Bioanalyzer 2100 system (Agilent Technologies, Santa Clara, CA, United States).

### RNA-Seq Library Construction and Sequencing

Approximately 1 μg of RNA per sample was used for the RNA sample preparations. Sequencing libraries were generated using NEBNext^®^ Ultra^TM^ RNA Library Prep Kit for Illumina^®^ (NEB, United States) following manufacturer’s recommendations and index codes were added to attribute sequences to each sample. In simple terms, RNA fragments are reversely transcribed and amplified into double-stranded cDNA, which is then connected to a NEBNext adapter with a hairpin ring structure. PCR products were purified by magnetic bead method and library quality was evaluated on Agilent Bioanalyzer 2100 system. Sequencing were performed on Illumina HiseqTM4000.

### RNA-Seq Data Analysis

In order to ensure the quality of the data for the following analysis, the raw data of nine samples were firstly processed through in-house perl scripts. In this step, clean data were obtained by removing reads containing adapter, reads containing ploy-N and low quality reads from raw data. At the same time, Q20, Q30, and GC content the clean data were calculated. All the downstream analyses were based on the clean data with high quality.

### Differentially Expressed Genes (DEGs)

The differential expression of C/LPS and LPS/HOA genes was analyzed by DESeqR package (1.18.0). DESeq provides a model for detecting negative binomial distribution to determine the expression of differentially expressed genes between each two groups.

The *p*-value of the result was assigned to each gene, carramine and Hochberg were used to control the error detection rate. The differential expression genes were screened under the conditions of fold change (FC) ≥ 2 and FDR < 0.05.

### GO and KEGG Pathway Enrichment Analysis of Differentially Expressed Genes

The DEGs were referred to the DAVID^1^ v6.7 for gene ontology (GO) and pathway analysis. GO terms with corrected *p*-value less than 0.05 were considered significantly enriched by DEGs. Pathway enrichment was determined using the Kyoto Encyclopedia of Genes and Genomes (KEGG) Pathway annotation. Pathways were considered enriched with *p* < 0.05.

### Reverse-Transcribed and Quantitative PCR (RT-qPCR)

To validate the RNA sequencing data, 15 differential expression genes in the Control group, LPS-induced group and HOA pre-treated group were selected for RT-qPCR analysis. cDNA was synthesized as described previously (Shen et al., 2017). A 20 μL PCR reaction mixture was prepared using a SYBR Green PCR Kit (Bio-Rad, Foster City, CA, United States) with four micrograms of cDNA as a template. After mixing, the PCR reaction was performed using CFX-96^TM^ Real-Time instrument (Bio-Rad, Foster City, CA, United States). The GAPDH gene was used as a house keeping gene to normalize the expression level of the test gene, and the relative gene expression level was analyzed using the 2^–ΔΔ^ ^CT^ CT method. All of the primers were synthesized by Sangon Biotech (Shanghai, China) and were listed in Table 1. All of the samples were analyzed in triplicate.

**TABLE 1:** Primer sequences of real-time qPCR assay.

Gene name	Forward primer (5′-3′)	Reverse primer (5′-3′)
Saa3	TGCCATCATTCTTTGCATCTTGA	CCGTGAACTTCTGAACAGCCTG
Bcl2l1	ATGTCTCAGAGCAACCGGGAGCT	TCACTTCCGACTGAAGAGTGAGCC
Mapkapk2	GGGCACCATGCTGTCGGGCTC	CGAGACACTCCATGACAATCAGC
Ccl9	AACAGAGACAAAAGAAGTCCAGAG	CTTGCTGATAAAAGATGATGCCC
Sdc4	GGGCAAGAAACCCATCTACAAA	CTCCAC TCCTCTCCCCAATAAGT
Ddx3x	CTCCGATTTCTCGGTACTCT	GACTTCCCTCTTGAATCACC
Socs3	CACAGCAAGTTTCCCGCCGCC	GTGCACCAGCTTGAGTACACA
Prdx5	TGGCAGAGCTGTTCAAGGGCAAGAA	TCAGCCAGGAGCCGAACCTTGCCTTC
TLR4	TCAGCAAAGTCCCTGATGACATTCC	AGAGGTGGTGTAAGCCATGCCA
Lif	GCTATGTGCGCCTAACATGAC	CGCTCAGGTATGCGACCAT
IL15	GCTCTTACCTGGGCATTAAGTAATGAA	CGCATGCAGTCAGGACGTGTTGATG
Tnfaip3	AACCAATGGTGATGGAAACTG	GTTGTCCCATTCGTCATTCC
TET2	TGTTGTTGTCAGGGTGAGAATC	TCTTGCTTCTGGCAAACTTACA
TGF-β1	CTCCCGTGGCTTCTAGTGC	GCCTTAGTTTGGACAGGATCTG
CCl20	CGACTGTTGCCTCTCGTACA	AGGAGGTTCACAGCCCTTTT

### Data Analysis

Data were presented as the mean ± standard deviation (SD). The significance was analyzed between the two groups using Student’s *t*-test and multigroup comparisons were compared using one-way analysis of variance (ANOVA). *P*-values of less than 0.05 were considered significant.

## Results

### mRNA Data Generation and Gene Comparison

The HiSeq TM 4000 was used for sequencing. We obtained 27,805,302 (98.297%), 26,990,712 (98.76%), and 29,943,562 (98.67%) clean reads in the control, LPS, and HOA groups, respectively (Table 2). The proportions of Q30 in the three samples were more than 92%. Therefore, the quality of clean reads obtained was high, and the results met the analysis requirements for subsequent experiments. The gene comparison showed that the rates of clean reads from the control, LPS, and HOA groups relative to the reference genome were 90.77, 92.66, and 93.61%, respectively, indicating that the utilization rate of sequencing data was high.

**TABLE 2:** Data quality and reference sequence alignment analysis results.

Summary	Control	LPS	HOA
N	50415	73089	84576
	0.14%	0.27%	0.27%
Adapters	326343	180454	178983
	0.67%	0.58%	0.57%
Low quality	149190	78390	84079
	0.34%	0.29%	0.27%
Clean	27805302	26990712	29943562
	98.30%	98.76%	98.67%
Clean base	8.34G	8.10G	8.98G
GC (%)	52%	51.04%	51.52%
Q30 (%)	94.12%	92.81%	92.73%
Genome map rate of clean reads	90.77%	92.66%	93.61%
Unique match in genome mapped reads	85%	86.99%	87.77%
Multiple match in genome mapped reads	5.77%	5.67%	5.81%

### Response of Differentially Expressed Genes (DEGs) to LPS Induction and HOA Pre-treatment

**FIGURE 2:**
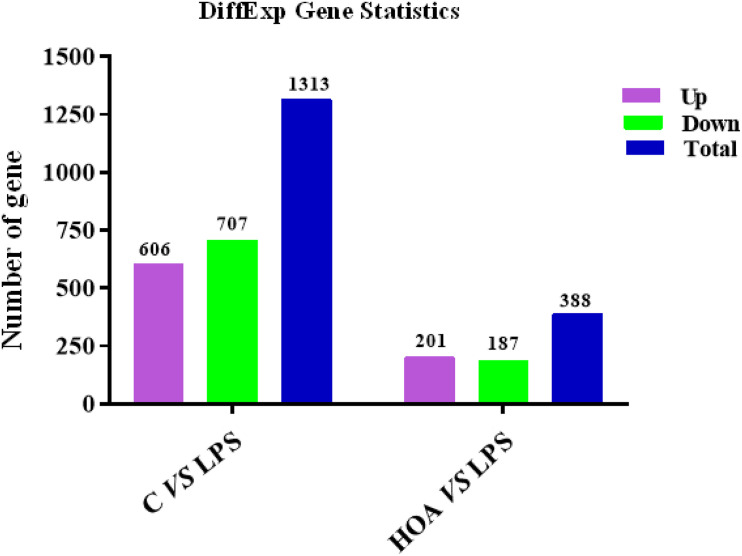
Distribution pattern for differentially expressed genes.

**FIGURE 3:**
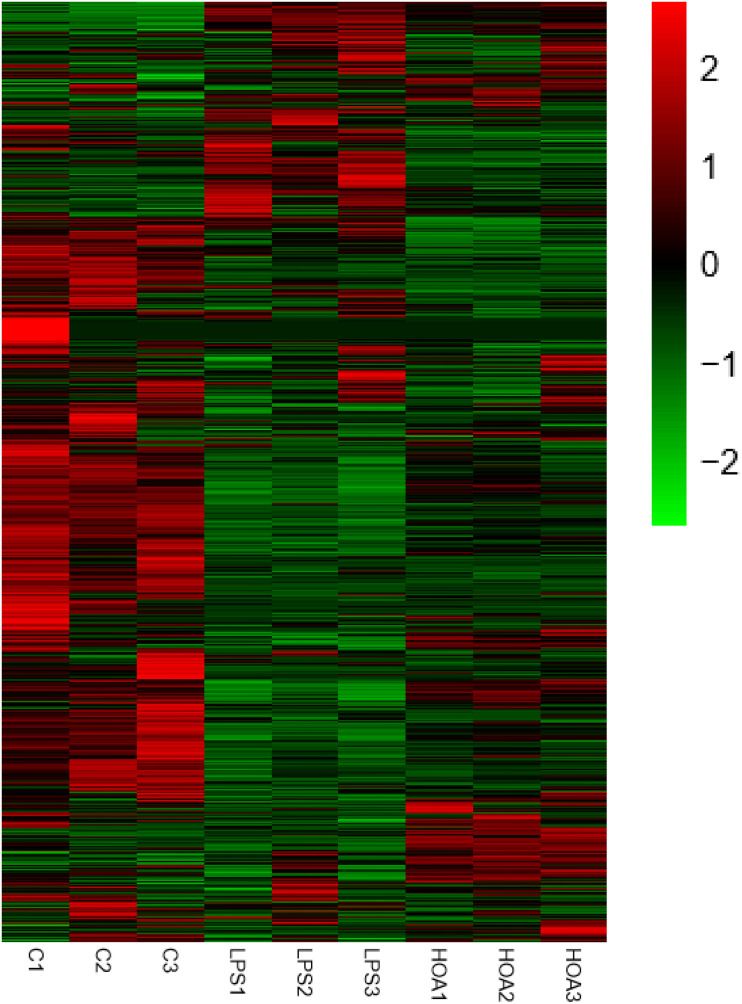
Differential gene expression change heat map. Red represents the upregulated genes, green represents the downregulated genes, and black represents no significant difference in genes expression.

The differential expression multiple between different samples was determined by the amount of gene expression. Using Log2 fold change ≥ 2 and FDR < 0.05 as criteria, a total of 1,313 DEGs were identified in the Control/LPS group, including 606 upregulated genes and 707 downregulated genes (Figure 1). In addition, 388 DEGs were identified in the HOA/LPS group, including 201 downregulated genes and 187 upregulated genes (Figure 2). DEGs identified in the biological replicates clustered together (Figure 3), indicating good reproducibility of treatments.

### GO Enrichment Analyses of DEGs

**FIGURE 4:**
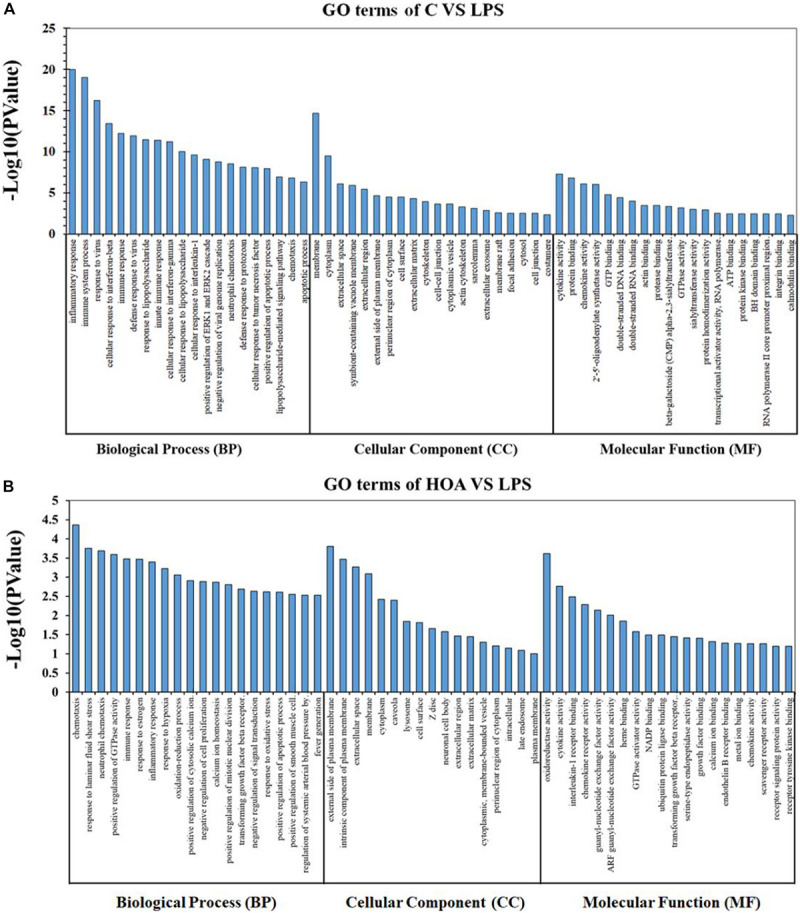
Gene Ontology (GO) classification for functional enrichment analyses with the differentially expressed genes (DEGs) in C vs. LPS **(A)** and HOA vs. LPS **(B)**. The top functional enriched classes of DEGs were annotated into three sub-ontology: Biological processes (BP), cell components (CC), and molecular function (MF).

The DEGs in the control/LPS and HOA/LPS groups were analyzed for GO functional enrichment (Figure 4). Each group of DEGs was annotated into three GO classifications: molecular function, cell components, and biological processes. In the biological process classification, the DEGs in the control/LPS group were mainly enriched in the inflammatory response, the immune system process, response to viruses, and the cellular response to interferon (IFN)-β. In the molecular function classification, the genes related to cytokine activity, protein binding, and chemokine activity significantly changed. In the cellular component classification, membrane, cytoplasm, and extracellular space changed significantly. After RAW 264.7 macrophages were pre-treated with HOA, the DEGs of the biological process classification in the LPS/HOA group were highly enriched in chemotaxis, response to laminar shear stress, positive regulation of GTPase activity and immune response. In the molecular function, the genes related to oxidoreductase, cytokine, and interleukin-1 receptor activity were significantly changed. In the cellular component classification, the external side of the plasma membrane, intrinsic components of the plasma membrane and extracellular space were significantly changed. These processes are involved in the inflammatory and immunomodulatory function of RAW 264.7 macrophages and provide a direction for the identification of DEGS in the future.

### KEGG Pathway Enrichment Analyses of DEGs

**FIGURE 5:**
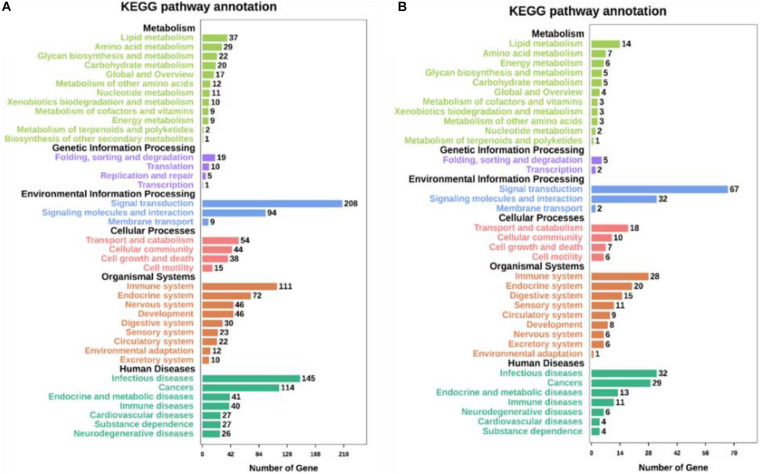
Kyoto Encyclopedia of Genes and Genomes (KEGG) pathways annotation and classification of DEGs in C vs. LPS **(A)** and LPS vs. HOA **(B)**.

**FIGURE 6:**
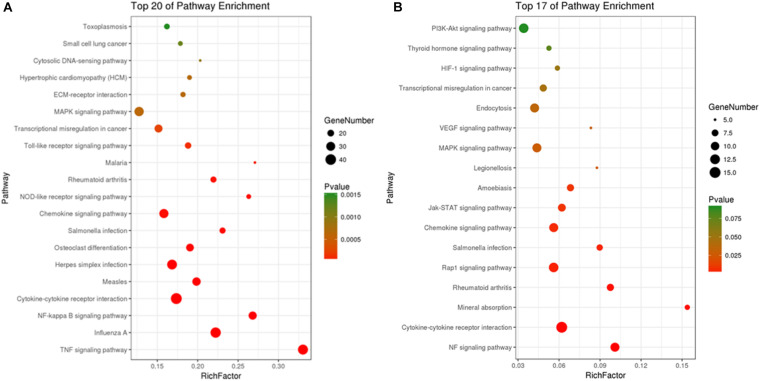
Scatter plot of KEGG pathways enrichment of DEGs in C vs. LPS **(A)** and LPS vs. HOA **(B)**.

Kyoto Encyclopedia of Genes and Genomes pathway analyses of the DEGs in control/LPS and LPS/HOA groups were performed using OmicShare. The DEGs of the two groups were mapped to the KEGG pathway database, and all pathways clustered into six categories: environmental information processing, human diseases, metabolic, organismal systems, cellular process, and genetic information processing. Environmental information processing mainly includes signal transduction, signal molecules and interaction, and membrane transport (Figure 5). A scatter plot was used to show the results of KEGG enrichment analyses. The top 20 signaling pathways enriched in the control/LPS group were biological processes related to inflammation, including the nuclear factor-kappa B (NF-κB) signaling pathway, TNF signaling pathway, toll-like receptor signaling pathway, chemokine signaling pathway, and NOD-like receptor signaling pathway (Figure 6A). In the LPS/HOA group, a total of 17 signaling pathways were enriched, in which NF-κB signaling pathway, cytokine-cytokine receptor interaction, Rap1 signaling pathway, chemokine signaling pathway, and the Janus kinase-signal transducer and activator of transcription proteins (JAK-STAT) signaling pathway were significantly enriched (Figure 6B).

### Differential Gene Annotation Into Immune-Related Metabolic Pathways

The DEGs of the control/LPS group and LPS/HOA group were analyzed using KEGG. The results showed that when RAW 264.7 cells were pre-treated with HOA for 30 min, the number of DEGs involved in the metabolic pathway of RAW 264.7 macrophages induced by LPS decreased significantly (Table 3). There were 939 and 151 DEGs involved in the pathway of the control/LPS group and LPS/HOA group, respectively. The number of annotated metabolic pathways in the HOA-treated group was significantly reduced, and there were 54 and 17 annotated metabolic pathways, respectively. By comparing the same inflammatory-related pathway between the two groups, the number of DEGs in the LPS/HOA group decreased in the same inflammatory immune-related pathway.

**TABLE 3:** Top 10 of KEGG pathways of C vs. LPS and HOA vs. LPS.

KEGG pathways of C vs. LPS	

Pathway	Count
TNF signaling pathway	36 (2.79%)
Influenza A	38 (2.95%)
NF-kappa B signaling pathway	26 (2.02%)
Cytokine-cytokine receptor interaction	42 (3.25%)
Chemokine signaling pathway	31 (2.40%)
Toll-like receptor signaling pathway	19 (1.47%)
Transcriptional misregulation in cancer	25 (1.94%)
MAPK signaling pathway	32 (2.48%)
PI3K-Akt signaling pathway	37 (2.86%)
Jak-STAT signaling pathway	19 (1.47%)
**KEGG pathways of LPS vs. HOA**	

**Pathway**	**Count (of 151)**

TNF signaling pathway	11 (1.89%)
Cytokine-cytokine receptor interaction	12 (3.95%)
Rheumatoid arthritis	13 (2.11%)
Rap1 signaling pathway	14 (3.16%)
Chemokine signaling pathway	15 (2.89%)
Jak-STAT signaling pathway	16 (2.37%)
MAPK signaling pathway	17 (2.89%)
VEGF signaling pathway	18 (1.32%)
Endocytosis	19 (2.89%)
Transcriptional misregulation in cancer	20 (2.11%)

### Differential Expression Level Analyses

**FIGURE 7:**
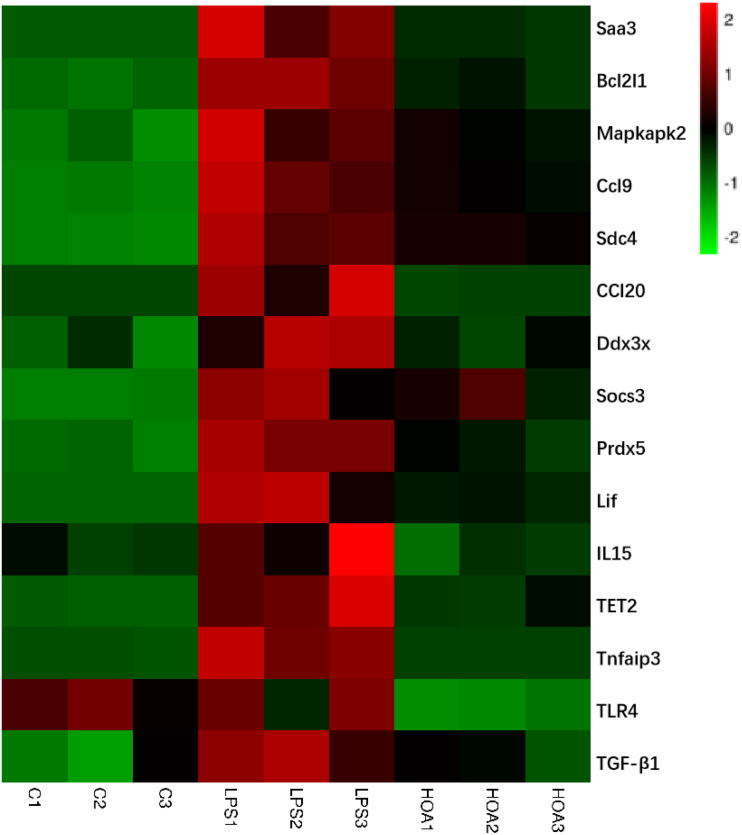
Heatmap of 15 representative immune-related DEGs in C vs. LPS and LPS vs. HOA. Red represents the genes expression were upregulated, green represents downregulated, and black represents no significant difference in genes expression.

Combined with the GO and KEGG enrichment results, 15 immune-related DEGs were selected for heat mapping (Figure 7). The results of thermography showed that immune-related genes such as Saa3, Bcl2l1, Mapkapk2, Ccl9, Sdc4, Ddx3x, Socs3, Prdx5, TLR4, Lif, IL15, Tnfaip3, TET2, TGF-β1, and CCl20 were significantly upregulated after LPS stimulation, but were downregulated in the HOA pre-treatment group.

### Verification of the RNA-Seq Data

**FIGURE 8:**
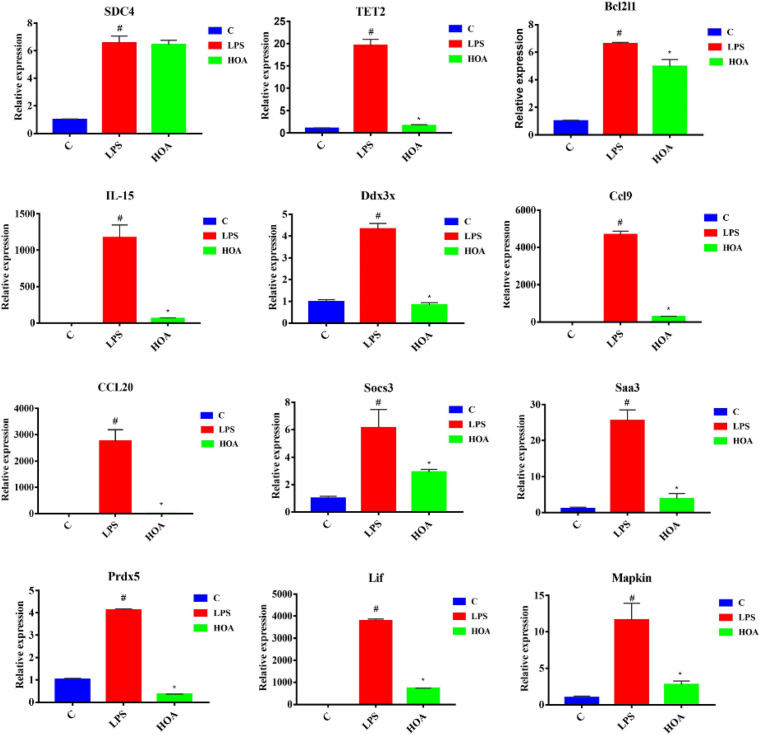
RT-qPCR Verification of fifteen differentially expressed genes. RAW 264.7 cells were pretreated with HOA (40 μM) for 30 min, and then stimulated with LPS (1 μg/mL) for an additional of 6 h. The data are presented as means ± SD (*n* = 3). *indicates a significant difference LPS vs. HOA (*p* < 0.05). ^#^indicates a significant difference C vs. LPS (*p* < 0.05).

To verify the accuracy of transcriptional sequencing data, we selected 15 DEGs associated with inflammation and immunity, including Saa3, Bcl2l1, Mapkapk2, Ccl9, Sdc4, Ddx3x, Socs3, Prdx5, TLR4, Lif, IL15, Tnfaip3, TET2, TGF-β1, and CCl20. RT-qPCR showed that the expression of these DEGs was increased (Figure 8), with similar trends as the RNA-seq samples, indicating that the sequencing results were reliable.

## Discussion

Lupane triterpenes have diverse pharmacological activities such as anti-inflammatory, anti-oxidation, anti-virus, anti-malaria, and immune regulatory effects (Sultana et al., 2003; Saleem, 2009). In addition, they also showed high anti-cancer activities including pancreatic cancer cells, breast cancer cells, melanoma, and prostate cancer cells (Hata et al., 2003; Saleem et al., 2009). Lupeol significantly decreases the expression of RAS protein in the human pancreatic cancer cell line ASPC-1, regulates the protein expression of protein kinase C, phosphatidylinositol 3′-kinase (PI3K)/Akt and mitogen-activated protein kinases (MAPKs), and significantly decreases the activation of the NF-κB signaling pathway (Murtaza et al., 2009). In a previous study, four triterpene acetates and four triterpene cinnamates isolated from the kernel fat of the shea tree effectively inhibited the 12-O-tetradecanoylphorbol-12-acetate-induced inflammation in mice. Lupeol cinnamate had the highest anti-inflammatory activity, including an obvious anti-inflammatory effect on carrageenan-induced paw swelling in rats (Akihisa et al., 2010).

To further prove the anti-inflammatory effects of HOA in macrophages, we also studied its effects on NO production in peritoneal macrophages induced by LPS. It inhibited the production of NO, was induced by LPS in a dose-dependent manner, and had no effect on cell activity, which further proves its anti-inflammatory activity (Cao et al., 2019).

The pathogenesis of inflammation is a complex process involving coordinated gene regulation. To understand the effects of HOA pre-treatment on the occurrence and development of inflammation in RAW 264.7 cells induced by LPS, RNA-seq was performed on normal cells, LPS-induced cells, and cells treated with HOA and LPS. There were significant differences in gene expression among the groups. HOA pre-treatment significantly inhibited the transcription of genes in RAW 264.7 cells induced by LPS compared to the LPS group (Figure 3), indicating that HOA pre-treatment might inhibit the activation and expression of related genes *in vitro*.

GO enrichment analyses indicated that DEGs in the control/LPS group were mainly enriched in the inflammatory response, immune system process, response to virus, and changes in IFN-β in cells (Figure 4A). This is consistent with the mechanism of action of LPS on macrophages (Mosser and Edwards, 2008). LPS stimulation activates macrophages to produce corresponding inflammatory and immune responses, and studies have shown that TLR4 endocytosis occurs in a dynamin-dependent manner after LPS stimulation, which induces IFN-β production. IFNs are proteins with immune activity, which can activate and regulate immune cells (Medzhitov, 2007). IFN interacts with specific receptors to activate STAT complexes. In the LPS/HOA group, the biological functions of DEGs mainly included chemotaxis, laminar shear stress response, and positive regulation of GTPase activity (Figure 4B). Inflammatory chemokines can modulate leukocytes (such as monocytes and neutrophils) from blood circulation to infection or tissue damage. In vascular endothelial cells, low levels of shear stress promote the secretion of endothelin, and endothelin-1 stimulates the release of other humoral inflammatory factors, cytokines, and leukocyte-releasing factors such as TNF-α and IL-6 (Pinho-Ribeiro et al., 2016). Therefore, the significant enrichment of genes in shear stress may inhibit LPS-induced inflammatory factor production in RAW 264.7 macrophages. Cytokines and inflammatory mediators produced by various stimuli can change the cytoskeleton of vascular endothelial cells (ECs), shrink ECs, and increase their permeability because cytokines and inflammatory mediators can activate GTPase through their corresponding receptors (Krishnamurti et al., 2002). These results suggest that GTPase may play an important role in vascular endothelial barrier dysfunction.

Kyoto Encyclopedia of Genes and Genomes pathway enrichment analyses showed that five inflammation-related pathways were screened from both the control/LPS group and LPS/HOA group at the same time, including the NF-κB signaling pathway, cytokine-cytokine receptor interaction, chemokine signaling pathway, the MAPK pathway, and the JAK-STAT signaling pathway (Figure 6). NF-κB is an important regulatory factor of proinflammatory gene expression. Local cells recognize pathogen-associated molecular patterns and release cytokines, which trigger an inflammatory cascade. Cytokines activate NF-κB and promote the localization and activation of macrophages in the infected site (Baker et al., 2010). Activated macrophages produce bacteriostatic molecules, release chemokines and cytokines, and promote macrophage activation and recruitment to damaged tissues. Bacteriostatic molecules work with aggregated white blood cells to kill pathogens and remove infected and dead cells. The NF-κB signaling pathway plays a key role in the development of inflammation-related metabolic diseases in the adipose tissue, central nervous system, and liver (Tornatore et al., 2012). Proinflammatory chemokines are produced by cells and have many biological functions, such as directional migration of leukocytes, inflammatory response, immune response, development and differentiation, and stimulation or inhibition of angiogenesis (Newton et al., 2016). The most important concern is the role of chemokines in inflammatory responses. It gathers white blood cells to the site of infection or injury and participates in leukocyte adhesion, migration, and chemotaxis. Chemokines prevent these cells from rolling and exudate through endothelial cells by inducing the expression of integrin in target leukocytes. Many chemokines have additional housekeeping functions in initiating an adaptive immune response and immune surveillance, and can activate JAK-STAT, MAPK, and protein kinase B pathways, thus regulating the inflammatory response. The JAK-STAT signaling pathway is used by many cytokines, and interferon and plays an important role in the development and function of innate and adaptive immunity (Croker et al., 2008). The JAK-STAT pathway acts as a central fulcrum in cell growth, differentiation, proliferation, and immunomodulation. Various growth factors, protein tyrosine kinases, and cytokines communicate through the JAK-STAT pathway to regulate gene transcription. In the pathogenesis of inflammatory diseases, many cytokines use JAK and STATs to transmit intracellular signals. In addition, MAPK signaling pathways are involved in intracellular inflammatory signaling cascades and are closely related to proinflammatory cytokines and NF-κB transcription activation (Zhou et al., 2016; Long et al., 2018). The MAPK signaling pathways are involved in a series of biological processes from inflammation, proliferation, differentiation, transformation, and apoptosis. These results suggest that LPS might stimulate macrophage activation through a variety of signaling pathways. Cytokine-cytokine interactions play an important role in immune and inflammatory responses. Interactions among proinflammatory cytokines (IL-1β, IL-6, IL-8, and TNF-α) play a synergistic role in cytokine production and cytokine activity. Anti-inflammatory cytokines such as IL-1Ra, IL-4, IL-10, and TGF-β1 play an antagonistic role in proinflammatory cytokines (Minciullo et al., 2016). Cytokine balance consists of two parts. The first is the balance in the cytokine system, such as IL-1 increasing the synthesis and secretion of IL-1RA, which may be upregulated by blocking the IL-1 receptor to weaken the harmful effects of IL-1. The other is the balance between different cytokine systems, such as TGF-β1 inhibiting the activities of IL-1 and TNF-α (Rubio-Perez and Morillas-Ruiz, 2012). In the LPS/HOA group, the number of inflammatory immune-related signaling pathways enriched by DEGs in the experimental group pre-treated with HOA decreased. Furthermore, the number of genes enriched in the same signaling pathway decreased, indicating that HOA pre-treatment of RAW 264.7 cells might inhibit LPS-induced activation of inflammatory-related signaling pathways in RAW 264.7 macrophages as well as the expression of some inflammatory-related genes (Table 3). To demonstrate that the DEGs obtained by transcriptome sequencing were more reliable, we selected 15 DEGs associated with inflammatory immunity using RT-qPCR to verify the sequencing results (Figure 8). The RNA-seq results showed consistent trends, demonstrating that RNA-seq sequencing data are reliable.

## Conclusion

We identified 15 significantly enriched GO terms and five typical pathways, suggesting that HOA may inhibit the inflammatory response induced by LPS in RAW 264.7 cells through a variety of targets and pathways. Additional studies are currently underway to prepare larger quantities of HOA and investigate its overall *in vivo* anti-inflammatory effect.

## Data Availability Statement

The original contributions presented in the study are included in the article/**Supplementary Material**, further inquiries can be directed to the corresponding author/s.

## Author Contributions

XY, JZ, YH, and YC: investigation. WH, LL, and YC: supervision and writing—review and editing. WH and YC: writing—original draft. All authors contributed to the article and approved the submitted version.

## Conflict of Interest

The authors declare that the research was conducted in the absence of any commercial or financial relationships that could be construed as a potential conflict of interest.

## Footnotes

1 http://david.abcc.ncifcrf.gov/home.jsp
